# A nitrogen-containing diphyllin derivative C156-P1 exhibited broad-spectrum antiviral activity against *Flaviviridae* viruses by preventing endosomal acidification

**DOI:** 10.1128/aac.00527-25

**Published:** 2025-09-26

**Authors:** Guoquan Chen, Wanfei Li, Ka Hei Lam, Mingyue Hu, Qian Wu, Xiangyu Xu, Yunzhu Huang, Fei Tang, Guohui Cui, Ping Cui, Jianping Zuo, Linna Liu, Jun Qian, Hong-Jie Zhang, Yi-Ping Li

**Affiliations:** 1 College of Animal Science and Technology, Guangxi University 622309, Nanning, China; 2 Department of Pathogen Biology and Biosecurity, Key Laboratory of Tropical Disease Control of Ministry of Education, Institute of Human Virology, Zhongshan School of Medicine, Sun Yat-sen University 26469 , Guangzhou, China; 3 School of Chinese Medicine, Hong Kong Baptist University, Kowloon, Hong Kong SAR, China; 4 First Affiliated Hospital of Jinan University 162698, Guangzhou, China; 5 Postdoctoral Research Center of Guangzhou Pharmaceutical Holdings Ltd., Guangzhou, China; 6 Life Science Institute, Guangxi Medical University, Nanning, China; 7 Laboratory of Immunopharmacology, State Key Laboratory of Drug Research, Shanghai Institute of Materia Medica, Chinese Academy of Sciences 58298 , Shanghai, China; 8 Institute of Infectious Disease, Guangzhou Eighth People's Hospital, Guangzhou Medical University, Guangzhou, China; IrsiCaixa Institut de Recerca de la Sida, Barcelona, Spain

**Keywords:** plant natural products, nitrogen-containing diphyllin derivatives, *Flaviviridae *viruses, dengue virus

## Abstract

Dengue virus (DENV) represents a significant public health threat, with its four serotypes estimated to account for approximately 96 million symptomatic infections annually. Currently, there are no antiviral agents available for the prevention or treatment of DENV infection. Here, we initially screened 12 diphyllin derivatives and identified C156-P1, a nitrogen-containing compound, as a potent agent against DENV infection. Further, C156-P1 exhibited inhibitory effects against the viruses of the *Flaviviridae* family, including four serotypes of DENV (DENV-1 to DENV-4) in multiple human and monkey cell lines, as well as Zika virus, Japanese encephalitis virus, yellow fever virus, and hepatitis C virus. In addition, C156-P1 also showed inhibition of the infections of herpes simplex virus type 1 and vesicular stomatitis virus, but not adenovirus and Sendai virus. Mechanistic studies demonstrated that C156-P1 inhibited DENV-2 after cell entry but before the endosomal membrane fusion step. C156-P1 inhibited vacuolar-type ATPase activity by perturbing the expression of ATP6V0A2 subunit, thereby suppressing endosomal acidification. Consequently, DENV was restricted in the late endosome, inhibiting virus fusion with endosomal membranes and resulting in infection inhibition. C156-P1 treatment also suppressed both IFN-I responses and endosomal TLR3 activation induced by DENV-2 infection. Furthermore, administration of C156-P1 in AG129 mice significantly reduced DENV-2 infection and effectively increased the survival rate of the mice. Taken together, our study demonstrates that the novel nitrogen-containing diphyllin derivative C156-P1 functions as a broad-spectrum antiviral agent by inhibiting endosomal acidification, thus representing a promising host-targeting antiviral candidate for future development.

## INTRODUCTION

Dengue virus (DENV) belongs to the *Orthoflavivirus* (formerly *Flavivirus*) genus of the *Flaviviridae* family, which also includes significant pathogenic viruses such as Zika virus (ZIKV), Japanese encephalitis virus (JEV), West Nile virus (WNV), and yellow fever virus (YFV) (1). DENV infections lead to a spectrum of diseases ranging from self-limiting dengue fever to severe dengue, a potentially lethal condition with hemorrhagic manifestations and capillary leak, formerly known as dengue hemorrhagic fever and dengue shock syndrome (2). Four DENV serotypes (DENV-1 to DENV-4) sustain the transmission cycle in humans through *Aedes* mosquitoes as vectors, leading to epidemics across 129 countries. This results in approximately 390 million infections annually, with around 96 million symptomatic cases and an estimated 10,000 fatalities (3–5). Furthermore, dengue was announced as one of the top 10 threats to global health by the World Health Organization in 2019. To date, no antiviral drug has been approved for treating DENV infection, and both DENV and other flaviviruses continuously pose significant threats to global health (6). Therefore, there is an urgent need for the development of anti-flavivirus drugs to treat flavivirus infections.

DENV is an enveloped virus with a single-stranded, positive-sense RNA genome of approximately 10.7 kilobases, comprising a single open reading frame (ORF) flanked by structured 5′ and 3′ untranslated regions (7). The ORF is translated into a polyprotein that is cleaved by cellular and viral proteases to produce three structural proteins (capsid, C; precursor membrane, prM; and envelope, E) and seven nonstructural proteins (NS1, NS2A, NS2B, NS3, NS4A, NS4B, and NS5) (8). The structural proteins constitute the virus particles and are critical for cellular entry, while the nonstructural proteins primarily facilitate viral genome replication and translation, as well as interactions with host factors essential for the viral life cycle (9). DENV is believed to enter host cells through clathrin-dependent receptor-mediated endocytosis (10). Internalized virions undergo low pH-dependent fusion of viral and endosomal membranes, releasing viral RNA into the cytoplasm, followed by translation in the endoplasmic reticulum (ER) and replication within invaginated membrane vesicles (11). After viral RNA is associated with C protein, a nucleocapsid is formed and coated with prM and E proteins before budding into the ER lumen, resulting in immature viral particles. These particles are transported through the Golgi to the trans-Golgi network, where they mature via prM cleavage by furin and are released (8, 12). Targeting distinct stages of the viral life cycle represents a fundamental strategy in drug development. Furthermore, the similar life cycles of flaviviruses enhance the potential for developing broad-spectrum anti-flavivirus drugs.

Numerous chemical compounds with therapeutic activities have been derived from natural products, predominantly from plants (13). Previously, we identified diphyllin (DP), an arylnaphthalene lignan (ANL), from the traditional Chinese medicinal plant *Justicia gendarussa* (14, 15). DP has been determined as a potent vacuolar-type ATPase (V-ATPase) inhibitor, which inhibits lysosomal acidification (16, 17). Studies have provided evidence supporting the broad-spectrum antiviral activity of DP against severe acute respiratory syndrome coronavirus 2 (SARS-CoV-2) (18), human immunodeficiency virus type 1 (HIV-1) (19), influenza virus (20), and Rift Valley fever virus (RVFV) (21). Additionally, DP exhibits multiple biological activities against tumors (22), inflammation (23), oxidation (24), and bacteria (25). However, the further development of DP as an antiviral therapeutic agent has been hampered by its low aqueous solubility and inadequate antiviral activity, highlighting the pressing need for structural modification. Recent studies have shown that nitrogen-containing diphyllin derivatives exhibit stronger anti-Ebola virus infection activity than DP (26). Our recent study showed that nitrogen-containing DP derivatives generally provide superior inhibitory activities against DENV-3 replicon and infections of DENV-1–4 (27). However, these findings require further validation using authentic flaviviruses, and the molecular mechanisms underlying their antiviral activities remain to be elucidated.

In the current study, we evaluated 12 DP derivatives for their potential anti-DENV activities using authentic virus infection assays. Among these, the nitrogen-containing DP derivative C156-P1 was identified and demonstrated potent antiviral activity against DENV-1–4 infections *in vitro* and *in vivo*. Furthermore, C156-P1 exhibited antiviral efficacy against *Flaviviridae* viruses, including ZIKV, JEV, YFV, and hepatitis C virus (HCV), as well as other enveloped viruses such as HSV-1 and VSV. Mechanistic studies revealed that C156-P1 inhibited viral infection by targeting the ATP6V0A2 subunit of V-ATPase, thereby preventing endosomal acidification.

## RESULTS

### The DP derivative C156-P1 efficiently inhibited DENV-2 infection *in vitro*

**Fig 1:**
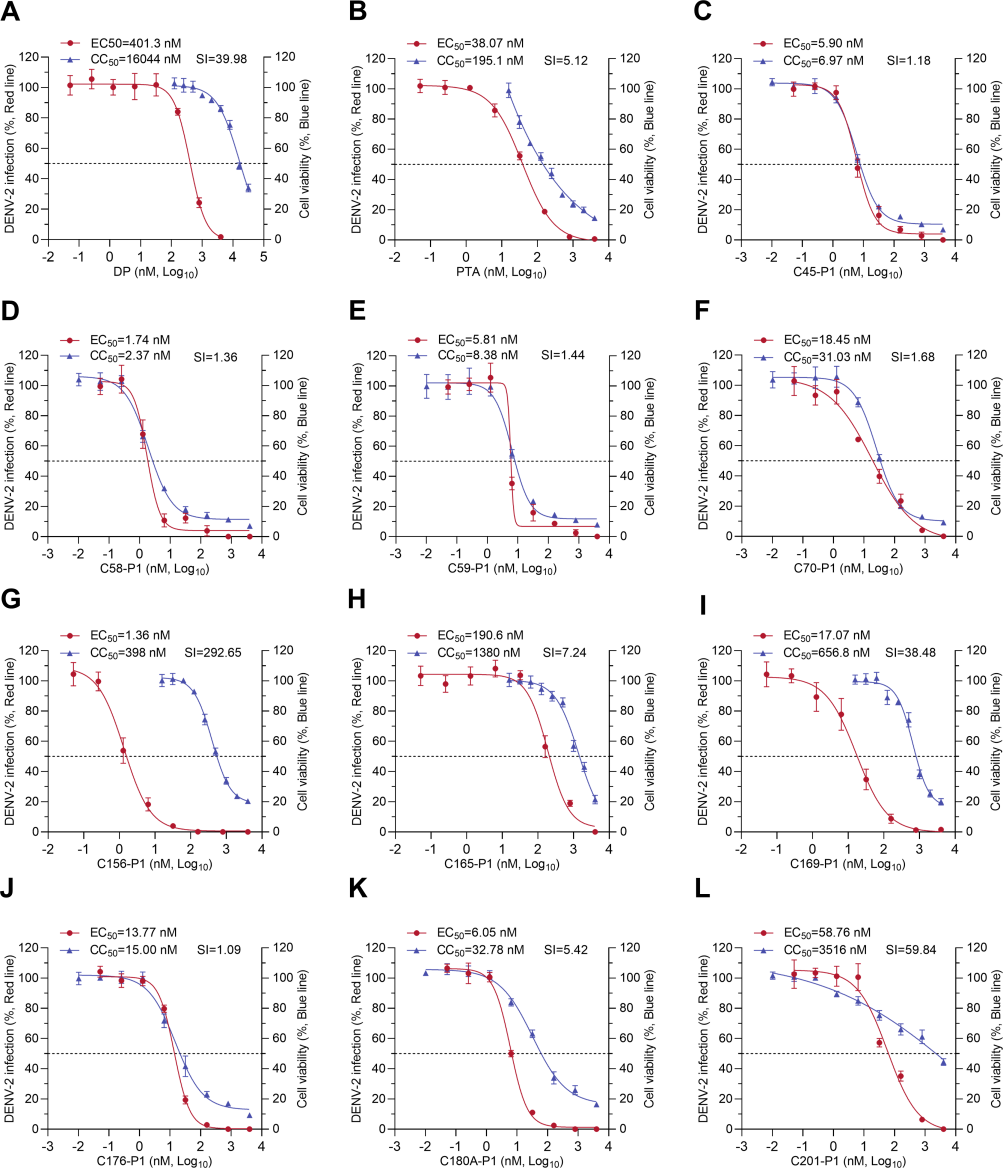
Nitrogen-containing arylnaphthalene lignans (*N*-ANLs) showed an enhanced inhibitory effect against DENV-2 infection compared to diphyllin. (**A–L**) A549 cells were infected with DENV-2 (MOI = 2) and incubated with DP, PTA, DP derivatives (C176-P1 and C180A-P1), or *N*-ANLs (C45-P1, C58-P1, C59-P1, C70-P1, C156-P1, C165-P1, C169-P1, and C201-P1) at the indicated concentrations for 48 h. The inhibition efficacy was depicted based on the results of an immunofluorescence assay. Cell viability was assessed using the CCK-8 assay 48 h post-treatment with DP, PTA, or an *N*-ANL, and data are presented as a percentage relative to DMSO-treated controls. Three independent experiments were performed for each compound, with triplicates included in all determinations. Data are shown as means ± SEM of three independent experiments. CC_50_: 50% cytotoxic concentration; EC_50_: 50% effective concentration; SI: selectivity index, the ratio of CC_50_ to EC_50_.

**Fig 2:**
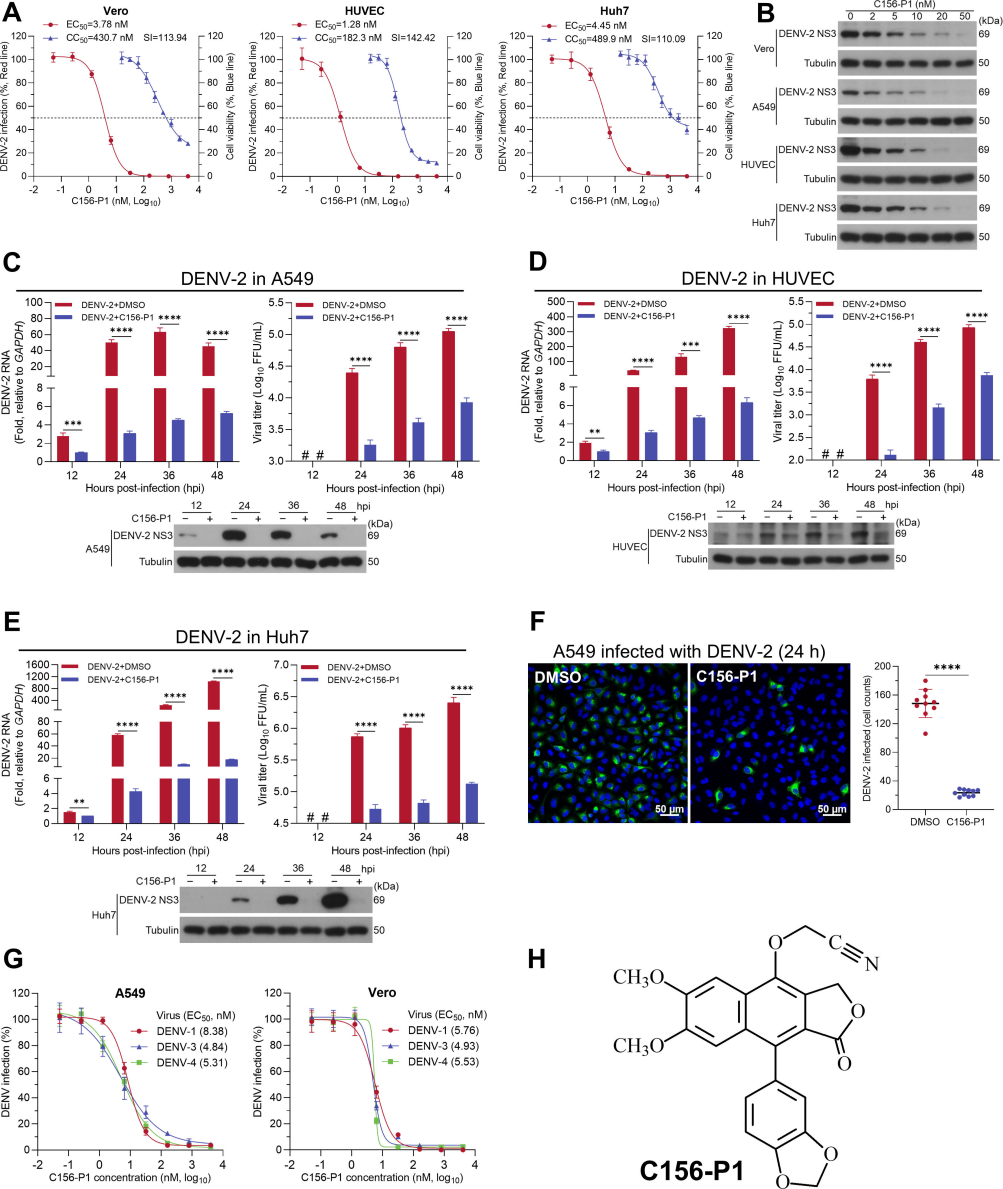
C156-P1 potently inhibited DENV infection in different cell lines. (**A**) C156-P1 inhibited DENV-2 infection in Vero, HUVEC, and Huh7 cells. Cells were infected with DENV-2 (MOI = 2) and incubated with C156-P1 for 48 h. The inhibition efficacy was depicted based on the results of an immunofluorescence assay. The cell viability was determined by the CCK-8 assay 48 h post C156-P1 treatment. Data are presented as a percentage in relation to DMSO-treated cells. (**B**) Vero, A549, HUVEC, and Huh7 cells were infected with DENV-2 (MOI = 2) for 48 h in the presence of various concentrations of C156-P1. The expression of DENV-2 NS3 protein was determined by Western blot, with Tubulin serving as the internal control. A549 (**C**), HUVEC (**D**), and Huh7 (**E**) cells were infected with DENV-2 (MOI = 2) containing DMSO or C156-P1 (50 nM). At 12, 24, 36, and 48 hpi, cells were collected, and the DENV-2 RNA (left panel), NS3 protein (bottom panel), and extracellular virus titer (right panel) were assessed using RT-qPCR, Western blot, and focus-forming assays, respectively. #, not detected. (**F**) A549 cells were infected with DENV-2 (MOI = 10) and treated with C156-P1 (50 nM) for 24 h. DMSO treatment was used as a negative control. DENV-2-positive cells were detected using an anti-DENV NS3 antibody, and nuclei were stained by Hoechst 33258. Scale bar: 50 µm (left panel). The quantification of DENV-2 infected cells was determined by ImageJ software (right panel). (**G**) A549 and Vero cells were infected with DENV-1, DENV-3, and DENV-4 at an MOI of 2, and incubated with C156-P1 at the indicated concentrations for 48 h. The inhibition efficacy was depicted based on the results of an immunofluorescence assay. Data are presented as a percentage in relation to DMSO-treated cells. (**H**) Chemical structure of C156-P1. In panels (C–F), data are processed by Student’s *t*-test and shown as means ± SEM of three independent experiments (**, *P* < 0.01; ***, *P* < 0.001; and ****, *P* < 0.0001).

The natural compounds DP and patentiflorin A (PTA, also known as 6-deoxyglucose-diphyllin) are ANL lead molecules isolated from the medicinal plant *J. gendarussa*, and they have shown promise as broad-spectrum antiviral agents (28, 29). We recently screened a library of 83 chemically synthesized ANL derivatives using a DENV-3 replicon and discovered that nitrogen-containing ANLs (*N*-ANLs) exhibited the highest antiviral potency (27). We selected 12 ANL derivatives with pronounced inhibitory effects on the DENV-3 replicon and evaluated their antiviral activities against the authentic DENV-2 strain 16681 (Fig. 1). The results showed that DP derivatives (C176-P1 and C180A-P1), nitrogen-containing DP derivatives (C156-P1, C165-P1, C169-P1, and C201-P1), and nitrogen-containing PTA derivatives (C45-P1, C58-P1, C59-P1, and C70-P1) exhibited lower 50% effective concentration (EC_50_) values against DENV-2 than their parent compounds (DP or PTA). However, nitrogen-containing derivatives of both DP and PTA also exhibited lower 50% cytotoxic concentration (CC_50_) in A549 cells compared to their parent compounds. Interestingly, the nitrogen-containing DP derivative C156-P1 showed the lowest EC_50_ value (1.36 nM) against DENV-2 and the highest selective index (SI = 292.65) value (Fig. 1G). The chemical structure of C156-P1 is shown in Fig. 2H, and the chemical structures of the other compounds are presented in Fig. S1. In summary, C156-P1 was identified as the most promising antiviral compound among ANL derivatives tested and was further explored in this study.

### C156-P1 inhibited the infection of four serotypes of DENV (DENV-1–4) in different cell lines

Subsequently, we assessed the antiviral efficacy of C156-P1 against DENV-2 in different cell lines. We found that C156-P1 inhibited DENV-2 infection in Vero, human umbilical vein endothelial cell line (HUVEC), and Huh7 cells, with EC_50_ <5 nM and SIs > 100 (Fig. 2A). The level of DENV-2 NS3 protein decreased with increasing concentrations of C156-P1 in Vero, A549, HUVEC, and Huh7 cells (Fig. 2B). A concentration of 50 nM C156-P1, which is tolerable for cytotoxicity (<62.5 nM in HUVEC cells), was used for subsequent experiments. We infected A549 cells with DENV-2 strain 16681 and observed that the viral RNA level and NS3 protein were downregulated in the C156-P1-treated groups in comparison with the DMSO-treated groups at 12, 24, 36, and 48 h post-infection (hpi). Concurrently, the viral titers were significantly decreased upon C156-P1 treatment at 24, 36, and 48 hpi (Fig. 2C). Similar inhibitory effects were observed in DENV-2-infected HUVEC and Huh7 cells (Fig. 2D and E). Immunofluorescence staining also showed a significant reduction in the number of DENV-2 NS3-positive A549 cells after C156-P1 treatment (Fig. 2F). Furthermore, C156-P1 effectively inhibited infections of DENV-1, DENV-3, and DENV-4 in A549 and Vero cells, with EC_50_ values ranging from 4.84 to 8.38 nM (Fig. 2G). Collectively, these results demonstrate that C156-P1 robustly suppresses DENV infection in multiple cultured cell lines in a dose-dependent manner.

### C156-P1 inhibited the infections of ZIKV, JEV, YFV, and HCV *in vitro*

**Fig 3:**
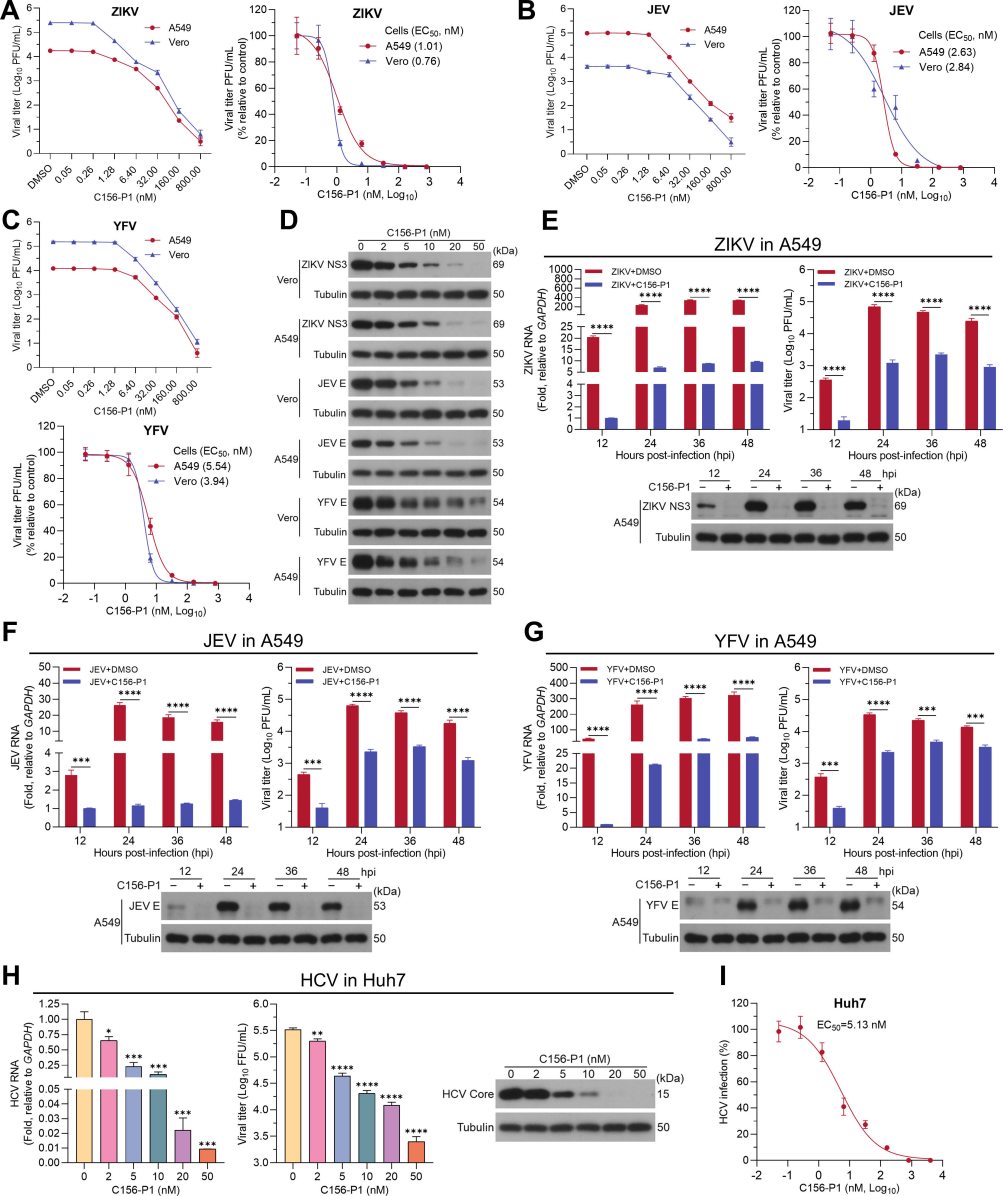
C156-P1 demonstrated broad-spectrum activities against *Flaviviridae* viruses. (**A–C**) A549 and Vero cells were infected with ZIKV (MOI = 2) (**A**), JEV (MOI = 2) (**B**), or YFV (MOI = 2) (**C**) and incubated with C156-P1 at the indicated concentrations for 48 h. Viral titers in the supernatants were measured by plaque assay, and the inhibition rates of virus infection were calculated. (**D**) Vero and A549 cells were infected with ZIKV (MOI = 2), JEV (MOI = 2), or YFV (MOI = 2) for 48 h in the presence of various concentrations of C156-P1. The expression levels of ZIKV NS3, JEV E, YFV E, and the internal control Tubulin were determined by Western blot. (**E–G**) A549 cells were infected with ZIKV (MOI = 2) (**E**), JEV (MOI = 2) (**F**), or YFV (MOI = 2) (**G**) containing DMSO or C156-P1 (50 nM). At 12, 24, 36, and 48 hpi, viral RNA (left panel), viral protein (bottom panel), and the viral titer (right panel) in the supernatants were detected by RT-qPCR, Western blot, and plaque assay, respectively. (**H**) Huh7 cells were infected with HCV (MOI = 0.5) and incubated with C156-P1 at the indicated concentrations for 72 h. HCV RNA levels, core protein expression, and the viral titers were detected by RT-qPCR, Western blot, and focus-forming assay, respectively. (**I**) Huh7 cells were infected with HCV (MOI = 0.5) and incubated with C156-P1 at the indicated concentrations for 72 h. The inhibition rates were calculated by immunofluorescence assays. In panels (E–H), data are presented as means ± SEM of three independent experiments (Student’s *t*-test; *, *P* < 0.05; **, *P* < 0.01; ***, *P* < 0.001; and ****, *P* < 0.0001).

To determine whether C156-P1 could inhibit other viruses of the *Flaviviridae* family, we examined the antiviral effects of C156-P1 on ZIKV (SZ01 strain), JEV (SA14-14-2 strain), YFV (17D strain), and HCV (JFH1 strain). In Vero and A549 cells, C156-P1 dose-dependently inhibited the infections of ZIKV, JEV, and YFV, with EC_50_ <6 nM for all three viruses, as determined by plaque assays (Fig. 3A through C). Consistently, the levels of ZIKV NS3, JEV E, and YFV E proteins decreased with increasing concentrations of C156-P1 in both Vero and A549 cells (Fig. 3D). Similar to its effects on DENV-2, C156-P1 significantly suppressed ZIKV (Fig. 3E), JEV (Fig. 3F), and YFV (Fig. 3G) infections, as evidenced by reductions in viral RNA, protein levels, and infectious virus titers in A549 cells at 12, 24, 36, and 48 hpi. In addition, we assessed the antiviral activity of C156-P1 against HCV in Huh7 cells and found that it markedly inhibited HCV infection in a dose-dependent manner (Fig. 3H), with EC_50_ value of 5.13 nM (Fig. 3I). These results suggest that C156-P1 exhibits broad-spectrum antiviral activities against multiple viruses within the *Flaviviridae* family.

### C156-P1 inhibited the infection of enveloped viruses via endocytosis entry

**Fig 4:**
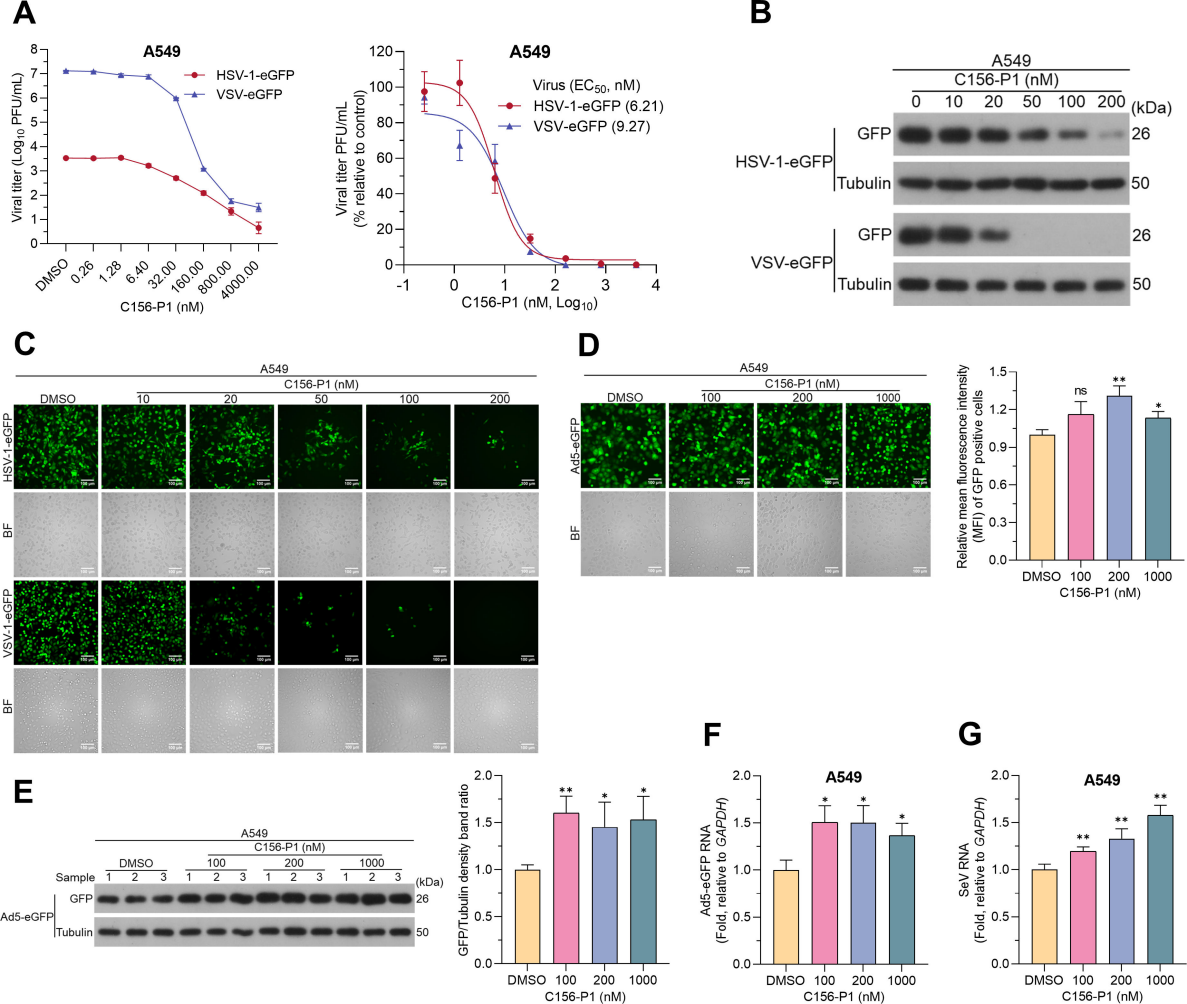
C156-P1 inhibited HSV-1 and VSV, but not AdV and SeV. (**A**) A549 cells were infected with HSV-1-eGFP (MOI = 0.01) or VSV-eGFP (MOI = 0.01) and incubated with C156-P1 at the indicated concentrations for 48 h or 24 h. The viral titers in the supernatants were determined by plaque assay (left panel), and the inhibition rates were calculated (right panel). (**B and C**) A549 cells were infected with HSV-1-eGFP (MOI = 0.01) or VSV-eGFP (MOI = 0.01) and incubated with C156-P1 at the indicated concentrations for 48 h or 24 h. The expression levels of GFP and tubulin were detected by western blot (**B**), and the fluorescence images were detected using fluorescence microscopy. Scale bars: 100 µm (**C**). (**D–F**) A549 cells were infected with Ad5-eGFP (MOI = 1) and incubated with C156-P1 at the indicated concentrations for 48 h. The fluorescence images were detected using fluorescence microscopy. Scale bars: 100 µm (D, left panel). The mean fluorescence intensity was calculated using ImageJ software (D, right panel). The intracellular GFP protein was detected by Western blot (E, left panel). Density of gel bands was analyzed (E, right panel). The levels of Ad5-eGFP RNA were detected by RT-qPCR (**F**). (**G**) A549 cells were infected with SeV (MOI = 0.1) and incubated with C156-P1 at the indicated concentrations for 24 h. The levels of SeV RNA were detected by RT-qPCR. In panels D-G, data are presented as means ± SEM of three independent experiments (Student’s *t*-test; ns, no significant; *, *P* < 0.05; **, *P* < 0.01).

To determine whether C156-P1 could inhibit other viruses beyond the *Flaviviridae* family, we tested its efficacy against viruses from the *Herpesviridae*, *Rhabdoviridae*, *Adenoviridae*, and *Paramyxoviridae* families. First, we evaluated the inhibitory efficacies of C156-P1 against HSV-1-eGFP and VSV-eGFP in A549 cells and found that the virus titers released to the supernatant decreased in a dose-dependent manner with C156-P1 treatment (Fig. 4A, left panel), with EC_50_ values of 6.21 nM for HSV-1-eGFP virus and 9.27 nM for VSV-eGFP virus determined by plaque assays (Fig. 4A, right panel). The reduction in virus titers was further confirmed by the levels of eGFP (Fig. 4B) and the number of eGFP-positive cells (Fig. 4C). Next, we tested whether C156-P1 showed inhibitory effect against adenovirus (AdV). We infected A549 cells with Ad5-eGFP virus and found no significant difference in the number of Ad5-eGFP-positive cells between DMSO-treated and C156-P1-treated cultures (Fig. 4D, left panel). Surprisingly, the mean fluorescence intensity (MFI) within the cells slightly increased after treatment with C156-P1 (Fig. 4D, right panel), and the expression levels of eGFP (Fig. 4E) and viral *hexon* mRNA (Fig. 4F) were also increased. Finally, we tested whether C156-P1 inhibited SeV infection by determining the mRNA expression level of the viral *nucleocapsid protein* (*NP*) gene (the SeV without reporter gene). Similar to Ad5-eGFP virus, C156-P1 did not inhibit SeV infection, and the viral *NP* mRNA levels were slightly increased in a C156-P1 concentration-dependent manner (Fig. 4G). Taken together, these results indicate that C156-P1 has a broad-spectrum antiviral activity against several *Flaviviridae* viruses (DENV, ZIKV, JEV, YFV, and HCV) as well as HSV-1 and VSV, but does not inhibit the infection of AdV and SeV.

We hypothesized that this may be related to the different pathways through which different viruses enter host cells. Both HSV-1 and VSV are enveloped viruses that utilize a cell-entry pathway similar to that of *Flaviviridae* viruses, involving endocytosis and pH-dependent fusion (30, 31). In contrast, AdV is a non-enveloped virus that enters host cells through clathrin-mediated endocytosis. Unlike *Flaviviridae* viruses, the AdV partial capsid uncoats in the endosomes to release protein VI, which ruptures the endosomal membrane, allowing partially disassembled virions to enter the cytoplasm (32). SeV, an enveloped virus, enters the host cell by direct fusion with the cell membrane (33). Our previous studies demonstrated that DP can inhibit endosomal acidification, preventing the fusion of the viral membrane with the endosomal membrane and subsequent infection (28). Taken together, we speculate that the nitrogen-containing DP derivative C156-P1 may inhibit the infections of viruses that enter host cells through endocytosis and fusion in a pH-dependent manner.

### C156-P1 inhibited flavivirus infection at the early stages after cell entry

**Fig 5:**
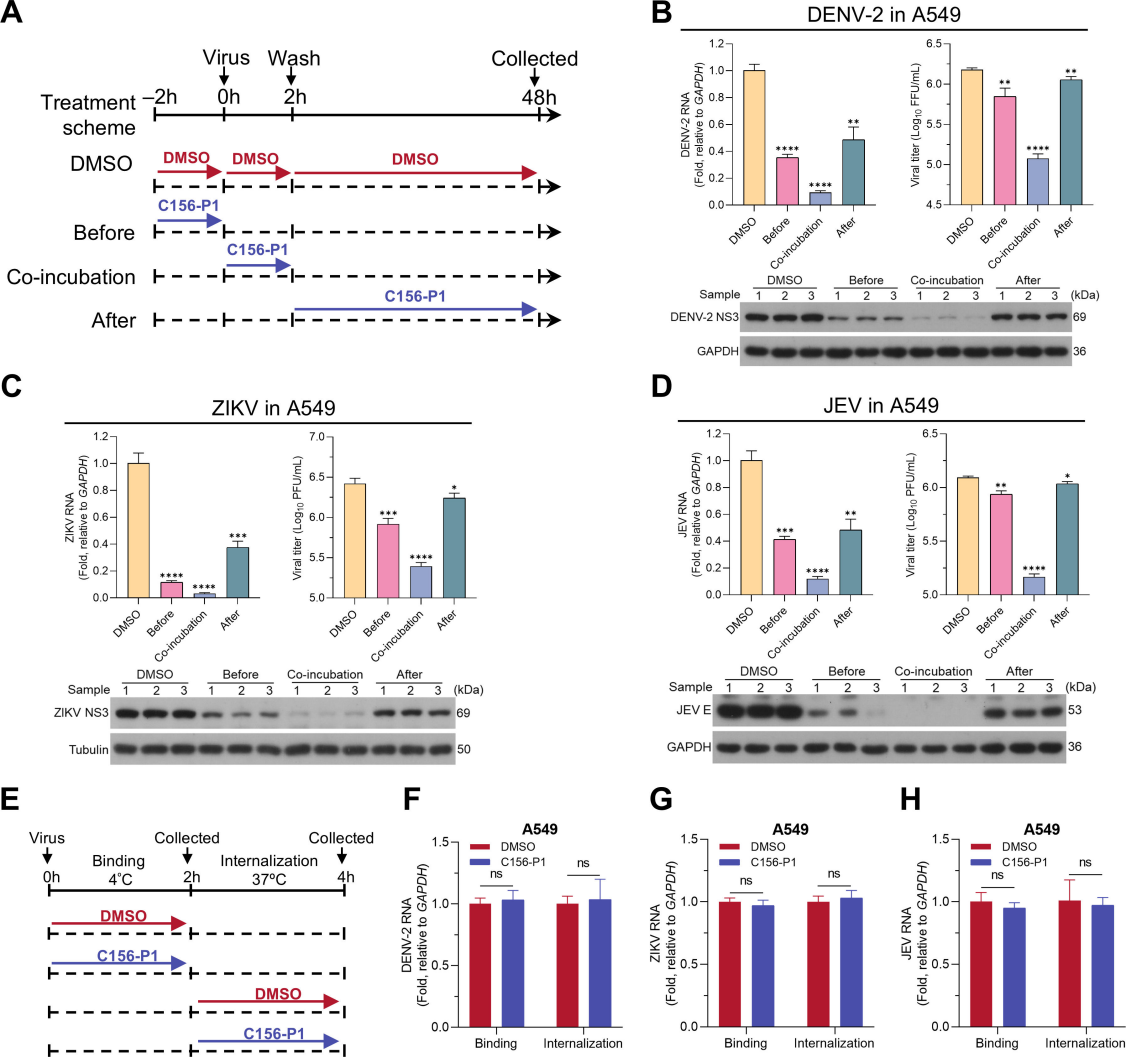
C156-P1 inhibited the infections of the DENV-2, ZIKV, and JEV at the early stages of their life cycles. (**A**) Schematic overview of the time-of-drug-addition assay. At time point 0 h, A549 cells were infected with DENV-2, ZIKV, or JEV at an MOI of 2. C156-P1 treatment was administered at three distinct times: from −2 to 0 h (before group), from 0 to 2 h (co-incubation group), and from 2 to 48 h (after group). The control group was treated with the same volume of DMSO. Cells and supernatants were harvested at 48 hpi for analysis. (**B–D**) The inhibitory effect of C156-P1 in each group was assessed by measuring viral RNA levels using RT-qPCR and viral protein expressions using Western blot. The infectivity titers of DENV-2 (**B**), ZIKV (**C**), and JEV (**D**) in the culture supernatants from panel A were determined using focus-forming and plaque assays. (**E**) Schematic overview of the binding and internalization assay. Experimental procedures were performed as described in the Materials and Methods section. (**F–H**) The RNA levels of DENV-2 (**F**), ZIKV (**G**), and JEV (**H**) during the binding and internalization phases were determined by RT-qPCR. In panels (B–D) and (F–H), data are presented as means ± SEM of three independent experiments (Student’s *t*-test; ns, no significant; *, *P* < 0.05; **, *P* < 0.01; ***, *P* < 0.001; and ****, *P* < 0.0001).

To determine which step of the viral life cycle might be targeted by C156-P1, we performed a time-of-drug-addition assay. C156-P1 was administered to A549 cells either at the virus entry (before and co-incubation) or post-entry (after) stage during flavivirus infection. A control group treated with the solvent DMSO was included in parallel (Fig. 5A). The cells and supernatants of each group were harvested at 48 hpi for subsequent analysis. In the DENV-2 infection experiment, the before-, during-, and after-incubation groups all demonstrated significantly reduced levels of intracellular viral RNA, viral NS3 proteins, and extracellular infectious virus titers compared to the DMSO control group (Fig. 5B). Among the three treatment groups, the co-incubation group showed the lowest levels of both viral RNA and infectious titers, followed by the before-incubation group and then the after-incubation group (Fig. 5B). Similarly, in ZIKV (Fig. 5C) or JEV (Fig. 5D) infections, the co-incubation group exhibited the lowest levels of infection. These results suggest that C156-P1 inhibits flavivirus infection at an early stage.

Next, we tested whether C156-P1 inhibited the early stages of flavivirus entry into host cells, including binding and internalization steps (Fig. 5E). The results demonstrated that C156-P1 did not exhibit inhibitory effects on either the internalization or binding steps of DENV-2 (Fig. 5F), ZIKV (Fig. 5G), and JEV (Fig. 5H) infections. Taken together, these results indicate that C156-P1 inhibits flavivirus infection at an early stage after the viruses have entered the cells.

### C156-P1 restricted the release of the DENV-2 genome into the cytoplasm from endosomes

**Fig 6:**
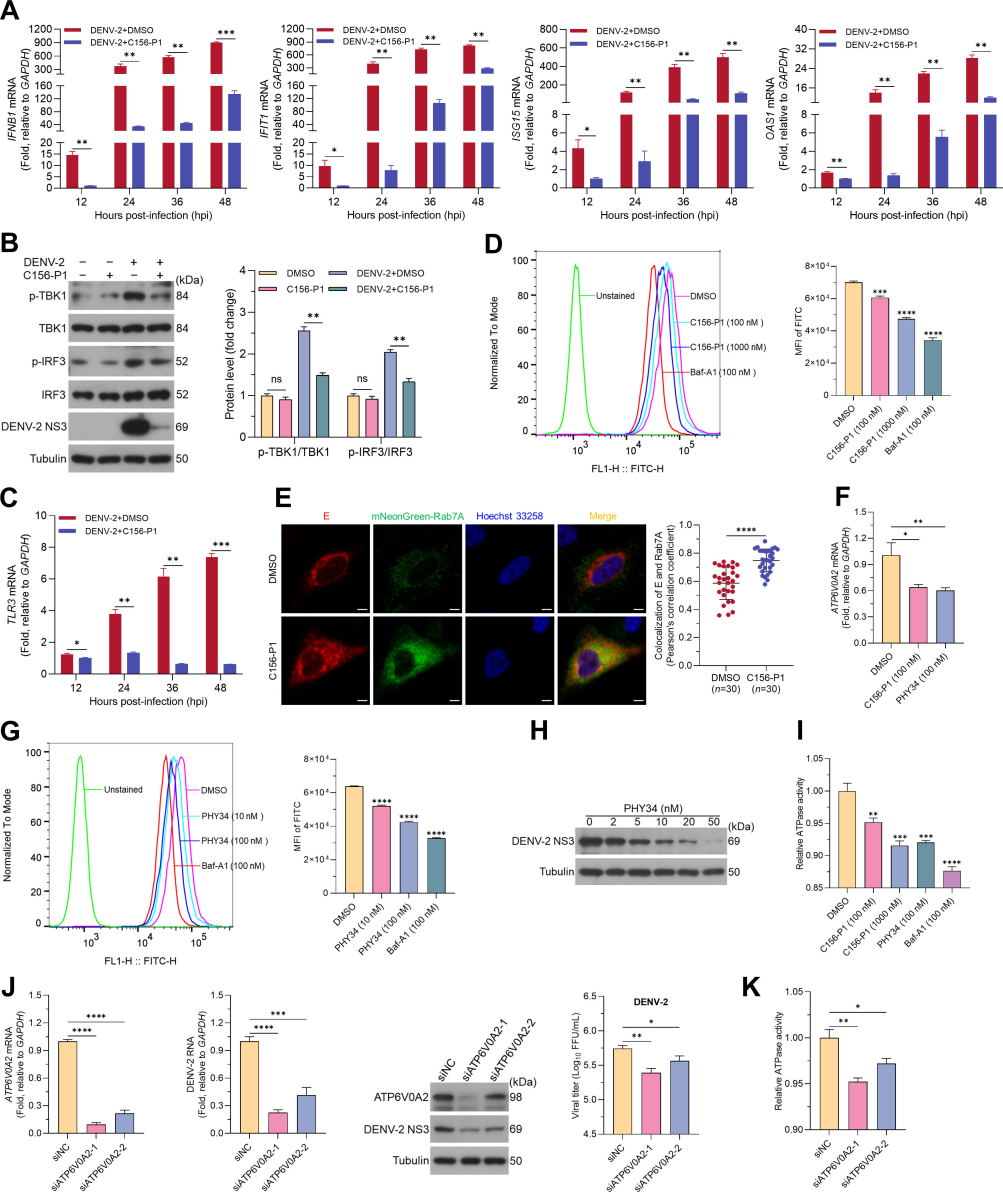
C156-P1 restricted DENV-2 release from the endosome into the cytoplasm by suppressing the ATP6V0A2 protein. (**A and C**) A549 cells were infected with DENV-2 (MOI = 2) and treated with C156-P1 (50 nM), and the cells were harvested at 12, 24, 36, and 48 hpi. The mRNA levels of *IFNB1*, *IFIT1*, *ISG15*, *OAS1*, and *TLR3* were quantitated by RT-qPCR. (**B**) A549 cells were infected with DENV-2 (MOI = 2) and incubated with C156-P1 (50 nM) for 24 h, after which the cell lysates were analyzed by Western blot (left panel), and band density analysis was performed (right panel). (**D**) A549 cells were pretreated with the indicated concentrations of C156-P1 or 100 nM bafilomycin A1 (Baf-A1) for 12 h, followed by incubation with 1 µM LysoSensor DND-189 for 30 min. The intensity of FITC staining was determined using a CytoFLEX flow cytometer (left panel), and the mean fluorescence intensity (MFI) was calculated (right panel). (**E**) A549 cells were transfected with plasmids expressing mNeonGreen-Rab7A for 24 h, followed by infection with DENV-2 (MOI = 20) and incubation with C156-P1 (50 nM) for 12 h. The cells were fixed and incubated with anti-DENV E primary antibodies, followed by Alexa Fluor 555-conjugated anti-Rabbit IgG secondary antibody for 1 h at room temperature. The representative images were acquired using the Zeiss LSM880. Scale bars: 5 µm (left panel). The colocalization of E and Rab7A proteins was analyzed using Pearson’s correlation coefficients with the JACoP plugin for ImageJ software. Data represent measurements using 30 cells from three independent experiments (right panel). (**F**) A549 cells were treated with DMSO, C156-P1 (100 nM), or PHY34 (100 nM) for 48 h, and *ATP6V0A2* mRNA levels were quantified by RT-qPCR. (**G**) A549 cells were pretreated with the indicated concentrations of PHY34 or 100 nM Baf-A1 for 12 h, followed by incubation with 1 µM LysoSensor DND-189 for 30 min. The intensity of FITC staining was determined by a flow cytometer (left panel), and the MFI was calculated (right panel). (**H**) A549 cells were infected with DENV-2 (MOI = 2) and incubated with PHY34 at the indicated concentrations for 48 h. The DENV-2 NS3 protein was determined by Western blot. (**I**) A549 cells were treated with DMSO, C156-P1 (100 nM or 1000 nM), PHY34 (100 nM), or Baf-A1 (100 nM) for 24 h, followed by the ATPase activity assay using 2 µg of total cell protein. (**J**) A549 cells were transfected with *ATP6V0A2*-specific or scramble siRNAs (NC) for 24 h, followed by infection with DENV-2 at an MOI of 2. The levels of *ATP6V0A2* mRNA, intracellular viral RNA, DENV-2 NS3 protein, and extracellular virus titer were assessed at 48 hpi. (**K**) A549 cells were transfected with *ATP6V0A2*-specific or scramble siRNAs (NC) for 48 h, followed by the ATPase activity assay using 2 µg of total cell protein. In panels (A–G) and (I–K), data are presented as means ± SEM of three independent experiments (Student’s *t*-test; ns, no significant; *, *P* < 0.05; **, *P* < 0.01; ***, *P* < 0.001; ****, *P* < 0.0001).

DENV infection triggers the type I interferon (IFN-I) response via host recognition of the released viral RNA in the cytoplasm (34, 35). DENV enters host cells through endocytosis, where low pH-induced conformational changes in the E protein facilitate virus fusion with the endosomal membrane, thereby releasing the viral genome into the cytoplasm (10). If DENV is inhibited at the pre-fusion stage by C156-P1, it is possible that viral RNA may not be released into the cytosol, or only a limited amount of viral RNA would be released. Thus, the IFN-I response may not be activated or may be attenuated in DENV-infected cells in the presence of C156-P1. To this end, we investigated the IFN-I response in A549 cells following DENV-2 infection with and without C156-P1 treatment. The results showed that DENV-2 infection significantly increased the IFN-I responses at 12, 24, 36, and 48 hpi, as determined by elevated mRNA levels of *IFNB1* and interferon-stimulated genes (ISGs) such as *IFIT1*, *ISG15*, and *OAS1* (Fig. 6A). Notably, the levels of *IFNB1* and these ISGs were apparently suppressed by C156-P1 treatment (Fig. 6A). Concurrently, the phosphorylation level of TANK-binding kinase 1 (TBK1) and IFN regulatory factor 3 (IRF3) was increased by DENV-2 infection, and this increase was also suppressed by C156-P1 treatment (Fig. 6B). As a control, C156-P1 treatment alone, without virus infection, did not change the levels of phosphorylated TBK1 and IRF3 (Fig. 6B). These results suggest that C156-P1 inhibits IFN-I responses induced by DENV-2 infection, implying that less viral RNA may be released into the cytoplasm.

Toll-like receptor 3 (TLR3) is a pattern recognition receptor (PRR) predominantly localized to the endosomal membrane, where it is primarily activated by viral double-stranded RNA (dsRNA) (36–38). Thus, the degree of TLR3 activation implies the sensing of viral dsRNA on the endosomal membrane. Here, we investigated the levels of TLR3 expression in A549 cells following DENV-2 infection and whether C156-P1 treatment modulated DENV-related TLR3 expression. The results showed that *TLR3* mRNA levels increased in a time-dependent manner at 12, 24, 36, and 48 hpi, which correlated with DENV-2 genome replication (Fig. 2C). As expected, C156-P1 treatment significantly reduced the expression of *TLR3* mRNA and abolished its time-dependent pattern (Fig. 6C). These results indicate that C156-P1 suppressed TLR3 activation, implying that dsRNAs were restricted from exposure to the endosomal membrane. Taken together, C156-P1 inhibited IFN-I responses and endosomal TLR3 activation induced by DENV-2 infection, suggesting that C156-P1 treatment largely restricts DENV release from endosomes into the cytoplasm.

### C156-P1 mediated endosomal acidification by targeting ATP6V0A2 subunit of ATPase to restrict DENV residing in endosomes

We have demonstrated that C156-P1 inhibited DENV infection during the early stages post-cell entry and reduced the activation levels of IFN-I and TLR3. This leads to the hypothesis that C156-P1 attenuates or suppresses the release of the viral RNA into the cytoplasm. It is known that following DENV entry into endosomes, endosomal acidification is a prerequisite for the conformational rearrangement of the DENV E protein, which prepares it for membrane fusion and viral RNA release. It is established that, following entry into the cell via endocytosis, DENV undergoes a conformational rearrangement of its E protein in response to endosomal acidification. This process facilitates the fusion of the viral and cell endosomal membranes, ultimately resulting in the release of the viral genome into the cytoplasm (39, 40). Therefore, we hypothesize that C156-P1 attenuates or suppresses the release of the viral genome into the cytoplasm, thereby reducing DENV infection.

Thus, we investigated whether C156-P1 inhibited DENV-2 infection by preventing endosomal acidification, as well as whether viral particles resided within the endosomes. First, we assessed whether C156-P1 affected the pH values of endosomes and lysosomes using an acid-sensitive indicator, DND-189 (41, 42). A V-ATPase inhibitor, bafilomycin A1 (Baf-A1), was used as a positive control to prevent the acidification of endosomes. The results demonstrated that C156-P1 (blue) and Baf-A1 (red) treatments shifted the peak of FITC leftward compared to the DMSO mock treatment (purple) (Fig. 6D, left panel). Besides, C156-P1 significantly decreased the MFI of FITC in a dose-dependent manner (Fig. 6D, right panel). These results indicate that C156-P1 exhibits a mechanism similar to Baf-A1 in preventing endosomal acidification.

After internalization, DENV particles are delivered to Rab5-positive early endosomes, which subsequently mature into Rab7-positive late endosomes, where virus-cell membrane fusion occurs in a low pH-dependent manner (43). To further determine whether C156-P1 restricts viral particles residing within the endosome, A549 cells were transfected with mNeonGreen-Rab7A, then infected with DENV-2 in the presence of C156-P1 (50 nM) for 12 h (DENV E proteins were not detectable at 2, 4, and eight hpi) (Fig. S2). Our findings revealed that C156-P1 significantly enhanced the colocalization between DENV E protein and Rab7A (Fig. 6E). These results indicate that C156-P1 inhibits endosomal acidification and restricts DENV particles within the endosomes.

Next, we sought to determine the mechanism underlying the endosomal retention of DENV induced by C156-P1. Previous studies have demonstrated that DP modulates the expression of V-ATPase, thereby inhibiting the acidification of endosomes (16, 28). Since C156-P1 induced endosomal acidification and restricted viral particles inside the endosomes, we proceeded to investigate whether V-ATPase played a role in this process. The V-ATPase complex comprises 32 subunits, and the ATP6V0A2 subunit plays a crucial role in endosomal proton transport, which is important for the function of V-ATPase (44, 45). Thus, we investigated whether ATP6V0A2 is involved in DENV infection and its correlation with C156-P1 treatment. We observed a decrease in *ATP6V0A2* mRNA expression following C156-P1 treatment of A549 cells, similar to the effect observed with PHY34 treatment, an inhibitor of ATP6V0A2 (Fig. 6F) (46). PHY34 treatment also inhibited endosomal acidification and DENV-2 infection in a dose-dependent manner, similar to C156-P1 treatment (Fig. 6G and H). We further examined whether C156-P1 and PHY34 affected V-ATPase activity. The results showed that C156-P1 and PHY34, similar to Baf-A1, significantly reduced V-ATPase activity. Additionally, the reduction of V-ATPase activity by C156-P1 was dose-dependent (Fig. 6I). Furthermore, siRNA knockdown of *ATP6V0A2* markedly decreased DENV-2 RNA levels, NS3 protein expression, and infectious virus titers (Fig. 6J). Knockdown of the ATP6V0A2 subunit also resulted in a reduction in intracellular V-ATPase activity (Fig. 6K). Taken together, these results suggest that the ATP6V0A2 subunit was a target of C156-P1 and is primarily responsible for preventing endosomal acidification, thereby inhibiting flavivirus infection.

### C156-P1 inhibited DENV-2 infection in mice

**Fig 7:**
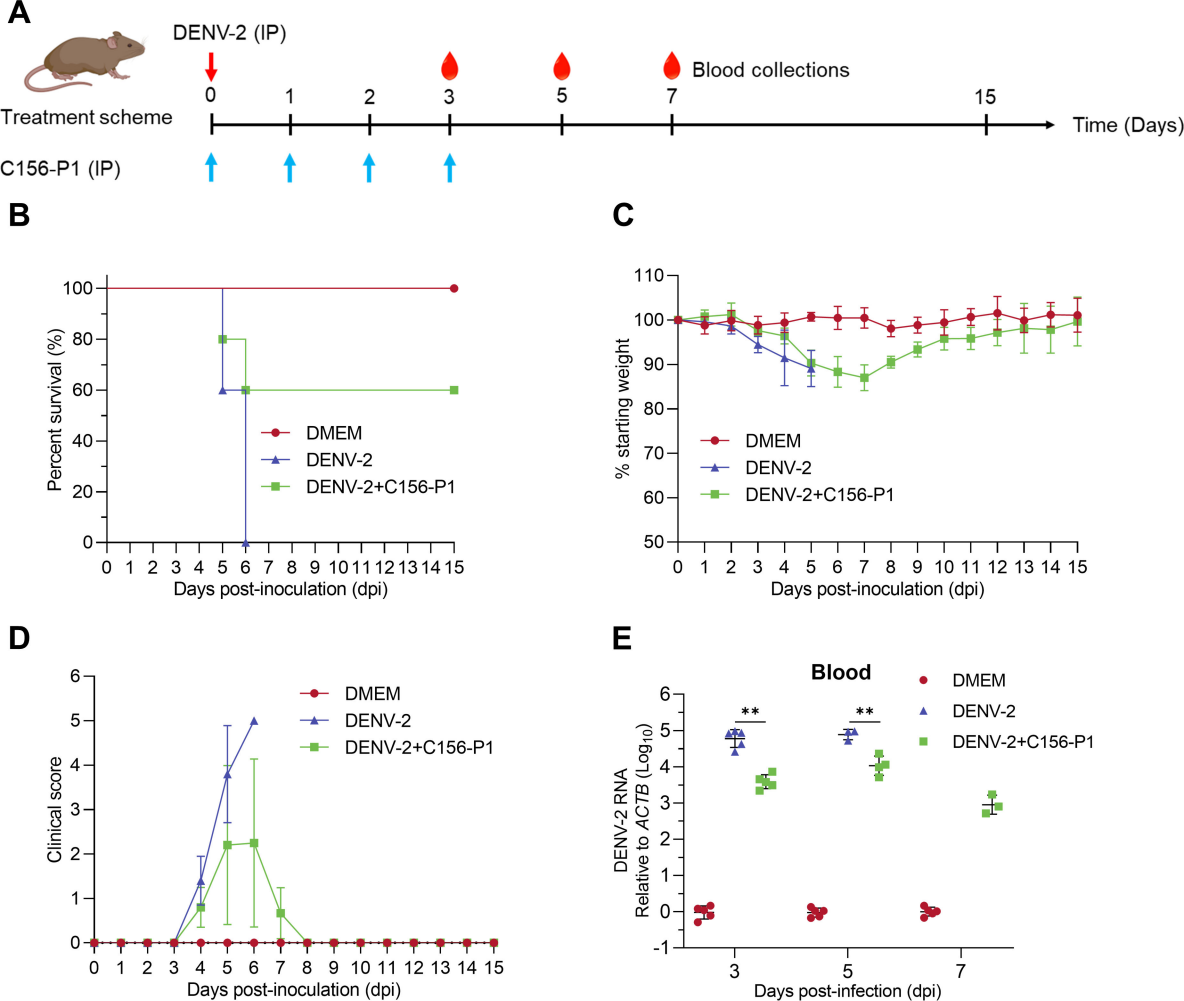
C156-P1 inhibited DENV-2 infection in AG129 mice. (**A**) Schematic of the experimental strategy for C156-P1 treatment in DENV-2-infected AG129 mice. AG129 mice (five mice per group) were inoculated intraperitoneally (i.p.) with DENV-2 (1 × 10^6^ PFU) or DENV-2 + 0.2  mg/kg of C156-P1. C156-P1 was injected i.p. to maintain the drug concentration *in vivo* for three consecutive days post-inoculation. An equal volume of DMEM (200 µL) was utilized as a negative control. (**B**) Survival curves of mice after DENV-2 infection. The experiment was kept for 15 days. (**C**) Mouse body weights are presented as a percentage of the body weight prior to inoculation, with standard deviations indicated. (**D**) Effects of C156-P1 on clinical symptoms in DENV-2-infected mice. (**E**) DENV-2 RNA levels in the blood were quantified using RT-qPCR at days 3, 5, and 7 post-infection. In panel (**E**), data are presented as means ± SEM. Statistical analysis was performed by Student’s *t*-test. **, *P* < 0.01.

To assess the therapeutic efficacy of C156-P1 on DENV infection, we infected AG129 mice, which are deficient in both IFN-α/β and IFN-γ receptors, with the DENV-2 strain 16681 and treated with C156-P1 (Fig. 7A). A dose of 0.2 mg/kg C156-P1 was selected for the mouse experiments based on dosage data from our previous animal studies with PTA in mice (28). We chose this dose because C156-P1 exhibited a lower EC_50_ value than PTA, and both C156-P1 and PTA are DP-derived derivatives. In the group not receiving C156-P1 treatment, a mortality rate of 100% was observed on day 6 post-virus challenge. In contrast, treatment with C156-P1 (0.2 mg/kg) protected the mice from this lethal dose challenge, resulting in a survival rate of 60% until the experiment was terminated on day 15 (Fig. 7B). In comparison to the DENV-2 group, treatment with C156-P1 mitigated the decline in body weight and enhanced behavioral signs in mice infected with DENV-2; the surviving mice began to recover from clinical symptoms from day 8 post-inoculation, and their body weight gradually recovered (Fig. 7C and D). None of the control group mice inoculated with DMEM exhibited any clinical symptoms or experienced weight loss. To further confirm the virus infection after injection, we determined the viral RNA loads in the blood samples (including blood cells) and found that C156-P1 markedly decreased DENV-2 in the blood of mice (Fig. 7E). These results indicate that C156-P1 exhibits therapeutic potential against DENV infection *in vivo*.

## DISCUSSION

There is currently no specific antiviral drug available for DENV infection, highlighting the urgent need to develop effective therapeutic agents for dengue. In this study, we discovered that C156-P1, a nitrogen-containing DP derivative, exhibited potent inhibitory effects against DENV-2 infection *in vitro* and *in vivo*. Furthermore, C156-P1 demonstrated broad-spectrum antiviral activities by inhibiting other serotypes of DENVs (DENV-1, DENV-3, and DENV-4) as well as other *Flaviviridae* viruses like ZIKV, JEV, YFV, and HCV. In addition, C156-P1 also inhibited the infections of enveloped viruses from other families, including HSV-1 and VSV. Mechanistic studies revealed that C156-P1 targeted the ATP6V0A2 subunit, affecting V-ATPase activity and suppressing endosomal acidification. This inhibitory mechanism restricted viral particles within the endosome, effectively blocking DENV infection both prior to and during the membrane fusion process. Collectively, these findings highlight the potentials of C156-P1 as a broad-spectrum antiviral agent, particularly against flaviviruses.

The natural compound DP is an ANL isolated from the *J. gendarussa* plant. DP functions as a V-ATPase inhibitor and is regarded as a promising broad-spectrum antiviral candidate. The compound was found to be active against SARS-CoV-2 (18), influenza virus (20), tick-borne encephalitis virus, WNV, ZIKV, RVFV, rabies virus, HSV-1 (21), Chikungunya virus (47), and VSV (48) at low micromolar concentrations. Although DP is recognized as a broad-spectrum antiviral agent, its limited aqueous solubility and insufficient antiviral activity considerably hinder its clinical applicability. To improve the antiviral activity and bioavailability of DP, various DP nanoparticles and structurally modified derivatives have been reported. DP nanoparticles exhibited reduced cytotoxicity and enhanced antiviral activity, as confirmed in influenza virus and feline infectious peritonitis virus (FIPV) (49, 50). Glycosylated DP derivatives, such as Justiprocumin B and PTA, have been shown to enhance antiviral activity, inhibiting HIV and ZIKV at nanomolar concentrations, respectively (28, 51). A recent study has found that various nitrogen-containing DP derivatives block Ebola virus entry at low nanomolar concentrations (26). From a series of 8 *N*-ANLs out of 12 tested ANLs, we identified C156-P1, which effectively inhibited DENV-2 infection at low nanomolar concentrations (EC_50_ < 5 nM), demonstrating antiviral efficacies *in vitro* across multiple monkey and human cell lines. The antiviral activity of C156-P1 against DENV-2 in A549 cells was nearly 300-fold greater compared to the parental compound DP. Notably, C156-P1 is structurally distinct from the other DP derivatives, as it is the only one containing a cyanide group. This nitrogen- and triple bond-containing cyanide group is positioned in close proximity to the DP skeleton. Although C70-P1 also has a cyanide group (Fig. S1), its cyanide group is located farther from the DP structure core. Compound 176-P1 has a structure very similar to C156-P1, but it contains only a carbon-carbon triple bond, rather than a cyanide group. The remaining compounds differ more significantly in structure from C156-P1. Furthermore, C156-P1 demonstrated potent antiviral effects against DENV-1, DENV-3, and DENV-4 infections at low nanomolar concentrations (EC_50_ < 10 nM), highlighting its strong anti-DENV activity.

Numerous anti-flavivirus compounds have been identified in recent decades; however, only a limited number have advanced to preclinical trials due to concerns regarding efficacy, specificity, toxicity, and stability (52). A recent study identified a potent DENV inhibitor, JNJ-1802, which blocks the NS3-NS4B interaction and generates effective anti-DENV infection effects in mice and rhesus macaques; a phase I first-in-human clinical trial has been completed for JNJ-1802 (53). However, JNJ-1802 lacks inhibitory effects against other *Flaviviridae* viruses. In contrast, C156-P1 is a broad-spectrum antiviral agent that not only potently inhibited DENV infection but also suppressed the infection of other *Flaviviridae* viruses. C156-P1 was demonstrated to effectively inhibit ZIKV, JEV, YFV, and HCV *in vitro* at low nanomolar concentrations (EC_50_ < 6 nM), significantly below the cytotoxic concentration. Chloroquine, an FDA-approved drug for treating malaria, can be used to prevent ZIKV infections by inhibiting endosomal acidification; however, the inhibitory concentrations necessary for ZIKV infection in cell culture are in the micromolar range (54, 55). The antiparasitic agent niclosamide can also inhibit DENV-2 infection by suppressing endosomal acidification, with an EC_50_ value of approximately 10 µM (56). In addition to inhibiting *Flaviviridae* viruses, C156-P1 could also inhibit viruses that enter host cells through either endocytosis or low pH-dependent fusion, as demonstrated for HSV-1 and VSV. In contrast, C156-P1 did not inhibit SeV, which enters cells not through the endocytosis pathway but by directly fusing with the host cell membrane. Similarly, C156-P1 did not inhibit AdV, which enters host cells by clathrin-mediated endocytosis without the requirement for fusion with the endosomal membrane. These findings further suggest that C156-P1 selectively inhibits viruses that enter host cells through an endocytosis pathway and undergo low pH-dependent fusion.

In the mechanistic study, we found that C156-P1 suppressed flavivirus infection in the early stages after viral entry into host cells, without affecting virus binding and internalization. Furthermore, C156-P1 reduced the activation of the IFN-I response and endosomal TLR3 activation induced by DENV infection. Therefore, these results indicated that the inhibitory effect of C156-P1 on DENV infection involves pathways other than IFN-I activation. An attenuated IFN-I response indicates that the IFN-I activator of DENV RNA was restrictedly exposed to the RNA sensor in the cytoplasm. Finally, we experimentally demonstrated that C156-P1 restricted DENV within the endosome, showing colocalization with Rab7A. Given that the early stages of the DENV life cycle after cell entry were inhibited, we found that C156-P1 targeted the pre-fusion stage or during the fusion of viral envelopes with endosomal membranes.

DP and PTA have been shown to inhibit the acidification of endosomes and lysosomes in a dose-dependent manner (28). C156-P1 is a nitrogen-containing derivative of DP, also demonstrated its ability to inhibit endosomal and lysosomal acidification in our study. DENV-2 enters host cells through clathrin-mediated endocytosis, delivering viral particles to Rab7-positive late endosomes for release into the cytoplasm via membrane fusion (43). Using confocal microscopy, we demonstrated that C156-P1 restricted DENV-2 to the late endosome. V-ATPase transports protons into the late endosome to maintain its acidic environment. HTP-013, a nitrogen-containing DP derivative, has been identified as a V-ATPase inhibitor that prevents lysosomal acidification by directly targeting ATP6V0A2 (17). Recent studies have shown that the loss of ATP6V0A2 expression significantly enhances the efficacy of HTP-013 in blocking EBOV infection (26). PHY34, structurally similar to DP, targets ATP6V0A2 and exhibits potent anticancer activity (46). We observed that both C156-P1 and PHY34 inhibited V-ATPase activity and downregulated *ATP6V0A2* mRNA expression, thereby suppressing DENV-2 infection. Moreover, C156-P1 inhibits V-ATPase activity in a dose-dependent manner. Therefore, the inhibition of V-ATPase activity through targeting the ATP6V0A2 subunit is the primary mechanism by which C156-P1 suppressed DENV-2 infection. Nevertheless, the mode of action of C156-P1 on ATP6V0A2 requires further investigation, such as through structural biology, chemical biology, and other complementary approaches. Altogether, our results indicate that C156-P1 inhibits V-ATPase activity by ATP6V0A2, thereby suppressing endosomal acidification and restricting DENV infection. *Flaviviridae* viruses (DENV, ZIKV, JEV, YFV, and HCV), *Herpesviridae* virus (HSV-1), and *Rhabdoviridae* virus (VSV) all enter cells via endocytosis and are transported to the acidic late endosome where they undergo membrane fusion to release the viral genome. Thus, C156-P1 exhibits its broad-spectrum antiviral activity through a common mechanism by inhibiting V-ATPase activity and thereby endosomal acidification, highlighting the potential of C156-P1 to inhibit other viruses that use the same entry strategy as *Flaviviridae* viruses.

Given the insensitivity of DENV-2 to wild-type mice, AG129 mice are commonly utilized to establish DENV-2 infection models (57). C156-P1 prevented DENV-2-induced mortality in AG129 mice when administered concurrently with the virus, suggesting its potential to inhibit DENV-2 infection *in vivo*. Chloroquine and hydroxychloroquine, the FDA-approved medications for the treatment of malaria, also inhibit ZIKV infection *in vivo* and prevent endosomal acidification. However, *in vivo* experiments indicate that chloroquine and hydroxychloroquine require inhibitory concentrations of 100 and 40 mg/kg in mice, respectively (55, 58). While further *in vivo* testing of C156-P1 is necessary, including dose optimization and drug formulation, our results suggested that C156-P1 effectively inhibited DENV-2 infection at a dosage of 0.2 mg/kg in mice.

In summary, we discovered that C156-P1, a novel nitrogen-containing DP derivative, exhibited broad-spectrum antiviral activities against five *Flaviviridae* viruses (DENV, ZIKV, JEV, YFV, and HCV) as well as two enveloped viruses (HSV-1 and VSV). C156-P1 targeted the ATP6V0A2 subunit to inhibit V-ATPase activity, thereby preventing endosomal acidification and restricting viral activity within the late endosome. Targeting a host pathway essential for a number of viruses allows C156-P1 with significant potential for future development as an agent against diverse viral infections.

## MATERIALS AND METHODS

### Cells and viruses

The human hepatoma cell line (Huh7) was provided by Dr. Charles Rice (Rockefeller University, New York, USA). The human adenocarcinomic alveolar epithelial cell line (A549), African green monkey kidney cell line (Vero), and baby hamster kidney cell line (BHK-21) were maintained in our laboratory (27, 59) and cultured in Dulbecco’s modified Eagle medium (DMEM; Gibco, 12800-017) with 10% fetal bovine serum (FBS; ExCell Bio, FSP500) and 1% penicillin-streptomycin (HyClone, SV30010). The HUVEC was provided by Dr. Yan Yuan (Sun Yat-sen University, Guangzhou, China) and cultured in Roswell Park Memorial Institute 1640 medium (Corning, 10-040-CVRC) supplemented with 10% FBS and 1% penicillin-streptomycin. All cells were grown at 37°C in a humidified incubator (Thermo Fisher Scientific, HERAcell 150i) with 5% CO_2_.

The DENV-2 strain 16681 clone was kindly provided by Dr. Andrew Yueh (National Health Research Institutes, Taiwan, China) (60). The DENV-1 clone D19044_7M, DENV-3 clone DV3syn_4M, and DENV-4 were developed in our laboratory (27). The ZIKV strain SZ01 and JEV strain SA14-14-2 were kindly provided by Dr. Chengfeng Qin (Beijing Institute of Microbiology and Epidemiology, Beijing, China) (61, 62). The YFV vaccine strain 17D was kindly provided by Dr. Ping Zhao (Naval Medical University, Shanghai, China) (63). The hepatitis C virus genotype 2a infectious clone JFH1 was kindly provided by Dr. Takaji Wakita (National Institute of Infectious Diseases, Tokyo, Japan) (64) and Dr. Charles Rice (Rockefeller University) (65). Sendai virus (SeV), vesicular stomatitis virus expressing enhanced green fluorescent protein (VSV-eGFP), and herpes simplex virus type 1 expressing eGFP (HSV-1-eGFP) were kindly provided by Dr. Jun Cui (Sun Yat-sen University) (66, 67). The adenovirus type 5 strain expressing eGFP (Ad5-eGFP) was kindly provided by Dr. Caijun Sun (Sun Yat-sen University) (68).

### Plasmid, antibodies, and reagents

Full-length human Rab7A cDNA was amplified by PCR. Rab7A cDNA was cloned into the pCDH-mNeonGreen expression plasmid to generate pCDH-mNeonGreen-Rab7A.

The primary antibodies used in this study were as follows: anti-DENV NS3 rabbit antibody (GeneTex, GTX124252), anti-DENV E rabbit antibody (GeneTex, GTX127277), anti-ZIKV NS3 rabbit antibody (GeneTex, GTX133309), anti-JEV E rabbit antibody (GeneTex, GTX125867), anti-YFV E rabbit antibody (GeneTex, GTX134024), anti-HCV Core C7-50 mouse antibody (Santa Cruz Biotechnology, sc-57800), anti-GFP tag mouse antibody (Proteintech, 66002-1-Ig), anti-horseradish peroxidase (HRP)-conjugated GAPDH antibody (Proteintech, HRP-60004), anti-HRP-conjugated Alpha Tubulin antibody (Proteintech, HRP-66031), anti-TBK1/NAK rabbit antibody (Abcam, ab40676), anti-phospho-TBK1/NAK rabbit antibody (Cell Signaling Technology, Ser172, D52C2), anti-IRF3 rabbit antibody (Cell Signaling Technology, D6I4C), anti-phospho-IRF3 rabbit antibody (Cell Signaling Technology, Ser396, 4D4G), and anti-ATP6V0A2 rabbit antibody (Abcam, ab96803). The secondary antibodies used for western blotting analysis were goat anti-mouse IgG (H + L)-HRP (Ray Antibody Biotech, RM3001) and goat anti-rabbit IgG (H + L)-HRP (Ray Antibody Biotech, RM3002). The secondary antibodies used for immunofluorescence were goat anti-rabbit IgG (H + L) highly cross-adsorbed secondary (Alexa Fluor Plus 488) (Invitrogen, A32731) and goat anti-rabbit IgG (H + L) highly cross-adsorbed secondary (Alexa Fluor Plus 555) (Invitrogen, A32732).

An ANL compound library was provided by Gihon Biotech Limited (Hong Kong, China). The chemical reagents bafilomycin A1 (Baf-A1, Selleck Chemicals, S1413), PHY34 (Targetmol, T37376), and LysoSensor Green DND-189 (Invitrogen, L7535) were purchased.

### Cell viability assay

The cells were seeded in 96-well plates at a density of 8 × 10^3^ cells/well and cultured at 37°C for 24 h. Serial dilutions of each compound, dissolved in 100 µL in DMSO/DMEM (2% FBS), were added to the cells. DMSO was used as a negative control. After incubation for 48 h, 10 µL of Cell Counting Kit-8 (CCK-8, Dojindo, CK04) was added to each well with gentle shaking, and the plates were incubated at 37°C for 30 min. The absorbance at 450 nm (OD_450_) was determined using a microplate reader (BioTek, ELX800). The 50% cytotoxic concentration (CC_50_) of a compound was calculated using GraphPad Prism 8.0 software (California, USA).

### Immunofluorescence and focus-forming units (FFUs) assay

The cells infected with DENV and HCV were analyzed by immunostaining. Briefly, virus-infected cells were fixed using methanol (−20°C) and incubated with primary antibody (anti-DENV NS3 or anti-HCV Core) at 4°C overnight, followed by incubation with secondary antibodies (conjugated with Alexa Fluor Plus 488) and Hoechst 33258 (Invitrogen, H1398) for 1 h at room temperature. Images were acquired using a fluorescence microscope (Leica DMI8, Germany), and the percentage of virus-positive cells was calculated by ImageJ software (National Institutes of Health, USA).

DENV infectivity titers were determined using a focus-forming assay as previously described (27). Briefly, Vero cells (8 × 10^3^ cells/well) were seeded into a 96-well plate and incubated for 24 h. The cells were inoculated with 10-fold serial dilutions of DENV in DMEM for 2 h. Subsequently, the virus inoculum was removed, and the cells were cultured with DMEM (2% FBS) for 48 h. The immunofluorescence assay was performed as described above. The number of FFUs was enumerated manually under a fluorescence microscope (Leica DMI8, Germany).

### Plaque assay

JEV infectivity titers were determined by plaque assay using BHK-21 cells, while ZIKV, YFV, HSV-1-eGFP, and VSV-eGFP infectivity titers were assessed using Vero cells. Briefly, Vero or BHK-21 cells were seeded in a 12-well plate for 24 h and inoculated with 10-fold serial dilutions of viruses in DMEM. After 2 h, the virus inoculum was removed, and fresh DMEM with 2% FBS and 1% methyl cellulose (Sigma-Aldrich, M0512) was added. After 4–5 days, the cells were fixed using 4% paraformaldehyde and stained with 1% crystal violet. The visible plaques were counted after washing the plates with tap water, and the virus infectivity titers were calculated and presented as plaque-forming units per milliliter (PFU/mL) of culture supernatant.

### Reverse transcription-quantitative PCR (RT-qPCR)

Total RNA was extracted from cells by use of MagZol reagent (Magen, R4801-02) according to the manufacturer’s instructions. For RT-qPCR, cDNA was synthesized using HiScript III RT SuperMix for qPCR Kit (Vazyme, R323-01) and subsequently amplified by qPCR using Magic SYBR Mixture reagent (CWBIO, CW3008M) with specific qPCR primers (Table 1). Relative mRNA expression of the interest genes was assessed using the 2^−ΔΔCt^ method and normalized to the *GAPDH* gene.

**TABLE 1:** The primers used for qPCR

Name^*a*^	Sense (5′−3′)	Antisense (5′−3′)
*hGAPDH*	GAAGGTGAAGGTCGGAGT	GAAGATGGTGATGGGATTTC
*hIFNB1*	CAGAAGGAGGACGCCGCATTGAC	CCAGGCACAGTGACTGTACTCC
*hIFIT1*	TTCGGAGAAAGGCATTAGA	TCCAGGGCTTCATTCATAT
*hISG15*	GCAACGAATTCCAGGTGTCC	GAGGTTCGTCGCATTTGTCC
*hOAS1*	GGCTGAAAGCAACAGTGCAG	GTGCAGGTCCAGTCCTCTTC
*hMX1*	CTCCGACACGAGTTCCACAA	GGCTCTTCCAGTGCCTTGAT
*hTLR3*	AAGGGTCTGTCTCACCTCCA	CTGAAAGCTGGCCCGAAAAC
*hATP6V0A2*	AAATGCAGGAGCAGTTGCAG	CCAGTTTTGCTCCCAGCCTC
*mACTB*	GGCTGTATTCCCCTCCATCG	CCAGTTGGTAACAATGCCATGT
DENV-2-*NS5*	AGTGCCCTTCTGTTCACACC	AGCTCCACATTTGGGCGTAA
ZIKV-*NS5*	CCGCGCCATCTGGTATATGT	TCCTTCCTCCTGGTATGCGA
JEV-*NS5*	TCAACGGAGTGGTGAAGCTC	TGGTCTCGTTGAGCACTTCC
YFV-*NS5*	AACAGGACGAGCTCATTGGG	CATGTTGTGCGTCCTTGTGG
HCV	CGGGAAGACTGGGTCCTTTC	ATCAGGCAGTACCACAAGGC
SeV-*NP*	CAGAGGAGCACAGTCTCAGTGTTC	TCTCTGAGAGTGCTGCTTATCTGTGT
Ad5-*hexon*	ACATTGGCTACCAGGGCTTC	GGGAAGTTAGCAGGGTAGGC

^*a*^ The prefix “h” denotes a human gene, whereas the prefix “m” signifies a mouse gene.

### Western blotting

The cells were harvested and lysed on ice for 30 min using lysis buffer (50 mM Tris-HCl, 150 mM NaCl, 1 mM EDTA, 1 mM DTT, and 0.5% TX-100, pH = 7.5), supplemented with a protease inhibitor cocktail (Sigma-Aldrich, P8849). The lysates were centrifuged at 4°C for 10 min at 12,000 rpm, and the supernatants were mixed with 1 × sodium dodecyl sulfate (SDS) loading buffer (Invitrogen, NP0008) and boiled for 10 min. Total protein was separated by 10% or 12% SDS-polyacrylamide gel electrophoresis (SDS-PAGE), and then transferred onto 0.2 µm polyvinylidene fluoride membranes (Bio-Rad, 1620177). The membranes were blocked with 5% skimmed milk for 2 h at room temperature and then incubated with the appropriate primary antibodies at 4°C overnight. After three washes with Tris-buffered saline supplemented with 0.1% Tween-20 (TBST) (10 min per wash), the membranes were further incubated with HRP-conjugated anti-mouse or anti-rabbit secondary antibodies for 1 h at room temperature. After three additional washes with TBST (10 min per wash), the protein bands were detected using enhanced chemiluminescence reagents (Proteintech, PK10001) and visualized with Fuji medical X-ray film (Fujifilm, RX-N-C).

### Time-of-drug-addition assay

A time-of-drug-addition assay was performed to evaluate the effect of C156-P1 on the steps of flavivirus life cycle. A549 cells seeded into 12-well plates were treated with C156-P1 (50 nM) either before, during, or after infection of DENV-2, ZIKV, or JEV. Briefly, in the before-incubation group, the cells were incubated with C156-P1 at 50 nM for 2 h, starting 2 h prior to infection (−2 h). After incubation, the cells were washed with phosphate-buffered saline (PBS) and then infected with DENV-2, ZIKV, or JEV at a multiplicity of infection (MOI) of 2 for 2 h. Finally, the virus inoculum was removed and replaced with fresh medium. In the co-incubation group, the cells were infected with the viruses in the presence of C156-P1 (50 nM) simultaneously (0 h). After 2 h, the incubation mixture was removed and replaced with fresh medium. In the after-incubation group, the cells were first infected with the viruses for 2 h (0 h). Following this, the virus inoculum was aspirated, and fresh medium containing C156-P1 (50 nM) was added. A control group treated with 0.1% DMSO was included in parallel, with treatment spanning from −2 to 48 h. All groups were incubated for 48 h. Viral RNA and proteins were subsequently analyzed using RT-qPCR and Western blot, respectively. The titers of viruses in the supernatants were quantified using either a focus-forming assay or a plaque assay.

### Viral binding and internalization assays

Viral binding and internalization assays were performed as previously described (69), with minor modifications. Briefly, A549 cells were incubated in C156-P1 or control DMSO from the time of virus infection to the end of the experiment. The cells were infected with DENV-2, ZIKV, or JEV at an MOI of 2 for 2 h at 4°C, and then washed three times with ice-cold PBS to remove unbound viral particles. For the binding assay, the cells were collected for RNA extraction and RT-qPCR to determine the RNA level of virions bound to the cell membrane. For the internalization assay, the cells were transferred to 37°C for 2 h. Subsequently, the cells were treated with proteinase K (1 mg/mL) for 3 min at room temperature to remove extracellular virus particles, followed by three PBS washes. Finally, the cells were collected for RNA extraction and RT-qPCR to determine the RNA level of virions internalized within cells.

### Flow cytometry

To detect the effect of C156-P1 on acid organelles through flow cytometry, bafilomycin A1 (Baf-A1), a V-ATPase inhibitor that prevents endosome acidification, was served as a positive control (70). A549 cells were pretreated with 100 nM or 1 µM C156-P1 for 12 h, and 100 nM Baf-A1 and 0.1% DMSO were used as positive and negative controls, respectively. Following pre-treatment, the cells were incubated with 1 µM LysoSensor DND-189 at 37°C for 30 min. The cells were then collected by generating a single-cell suspension, which was passed through a nylon net. Flow cytometry measuring the intensity of FITC using a flow cytometer (Beckman, CytoFLEX), and data analysis was performed using FlowJo software (BD Biosciences, USA).

### Confocal microscopy

For confocal microscopy analysis, 20 mm diameter cover glasses (WHB Scientific, WHB-12-CS) were placed in a 12-well plate, and then 8 × 10^4^ cells were seeded in each well and cultured for 18 h before virus infection. The cell cover glasses were fixed with 4% paraformaldehyde (Sangon Biotech, E672002) for 20 min at room temperature, permeabilization with 0.1% Triton X-100 in PBS for 10 min, and blocked with 3% bovine serum albumin for 1 h. The cells were incubated with primary antibodies (anti-DENV NS3 or E) for 2 h at room temperature, followed by secondary antibodies (conjugated with Alexa Fluor Plus 488 or 555) and Hoechst 33258 (Invitrogen, H1398) for 1 h at room temperature in the dark. Cell images were acquired with a confocal laser-scanning microscope (Zeiss, LSM880). The images were analyzed and processed using ZEN and Photoshop software. Pearson’s correlation coefficient was calculated using the JACoP plugin for ImageJ software (National Institutes of Health) (71).

### ATPase activity assay

ATPase activity assay was performed as described previously with minor modifications (72). Total cellular protein was extracted using a lysis buffer (Beyotime, P0013J) supplemented with a protease inhibitor cocktail (Selleck Chemicals, B14001). Proteins were quantified using a Bicinchoninic Acid Protein Assay Kit (Beyotime, P0010) according to the manufacturer’s instructions. ATPase activity was determined using 2 µg of cell lysate with the ATPase/GTPase Activity Assay Kit (Sigma-Aldrich, MAK113) according to the manufacturer’s instructions. Following a 30-min incubation and a 30-min termination at room temperature, the samples were measured with a microplate reader (BioTek, ELX800) at 630 nm (OD_630_).

### RNA interference

Small interference RNAs (siRNAs) targeting human *ATP6V0A2* gene and scrambled siRNA controls were designed and synthesized by GenePharma (Suzhou, China) (Table 2). The siRNAs were transfected into A549 cells for 6 h using GP-transfect-Mate reagent (GenePharma, G04008) according to the manufacturer’s instructions. At 48 h post-transfection, the A549 cells were harvested for further analysis.

**TABLE 2:** The siRNAs used in the study

Name	Sense (5′−3′)	Antisense (5′−3′)
si*ATP6V0A2-*1	GUGCUACAAUCCCCUCAUUTT	AAUGAGGGGAUUGUAGCACTT
si*ATP6V0A2-*2	CCGCAGAGCAUAAGAAGAUTT	AUCUUCUUAUGCUCUGCGGTT
Negative control	UUCUCCGAACGUGUCACGUTT	ACGUGACACGUUCGGAGAATT

### Animal experiments

All animal experiments were approved by the Institutional Animal Care and Use Committee of Sun Yat-sen University Guangzhou, China (approval number: SYSU-IACUC-2024-B1498). AG129 mice (129/Sv mice deficient in interferon-α/β and interferon-γ receptors) were from Prof. Jianping Zuo (Shanghai Institute of Materia Medica, Chinese Academy of Sciences, China) and maintained under specific-pathogen-free conditions at Sun Yat-sen University. Fifteen AG129 mice (6–8 weeks) were randomly divided into three groups. In the DENV-2 challenge group, five mice were inoculated with DENV-2 strain 16681 (1 × 10^6^ PFU) without any additional treatment. In the C156-P1 treatment group, five mice were inoculated with DENV-2 strain 16681 (1 × 10^6^ PFU) containing C156-P1 (0.2 mg/kg). C156-P1 was administered for three consecutive days following DENV-2 inoculation. In the mock infection group, five mice were injected with DMEM. Each injection was performed intraperitoneally with 200 µL per dose for each mouse. Mortality, symptoms, and body weight of all mice were monitored for 15 days. Clinical severity was scored as previously described (73). Briefly, the scoring system includes five grades, namely grade 0 (healthy), grade 1 (ruffled hair or hunchbacked), grade 2 (reduced mobility), grade 3 (limb weakness), grade 4 (limb paralysis), and grade 5 (moribund or death). Blood samples (including blood cells) were collected from mice via tail cutting to determine virus copies in blood at 3, 5, and 7 days post-inoculation (dpi).

### Statistical analysis

All experimental data were presented as mean and standard error of mean (mean ± SEM) from at least three independent experiments. Statistical analysis was performed using Student’s *t*-test with GraphPad Prism 8 software (California, USA), and *P* < 0.05 was considered significant.

## Data Availability

The data generated and analyzed in the present study are included in this manuscript and the supplementarl material. Additional information is available from the corresponding author upon reasonable request.

## SUPPLEMENTAL MATERIAL

Supplemental material aac.00527-25-s0001.docx Fig. S1 and S2.

ASM does not own the copyrights to Supplemental Material that may be linked to, or accessed through, an article. The authors have granted ASM a non-exclusive, world-wide license to publish the Supplemental Material files. Please contact the corresponding author directly for reuse.
