# Supplementary material for: A nitrogen-containing diphyllin derivative C156-P1 exhibited broad-spectrum antiviral activity against Flaviviridae viruses by preventing endosomal acidification
Source: Antimicrob Agents Chemother. 2025 Sep 26;69(11):e00527-25. doi: 10.1128/aac.00527-25 (PMC12587552; doi:10.1128/aac.00527-25)
Supplement: Supplemental material — Fig. S1 and S2. [file aac.00527-25-s0001.docx]

**
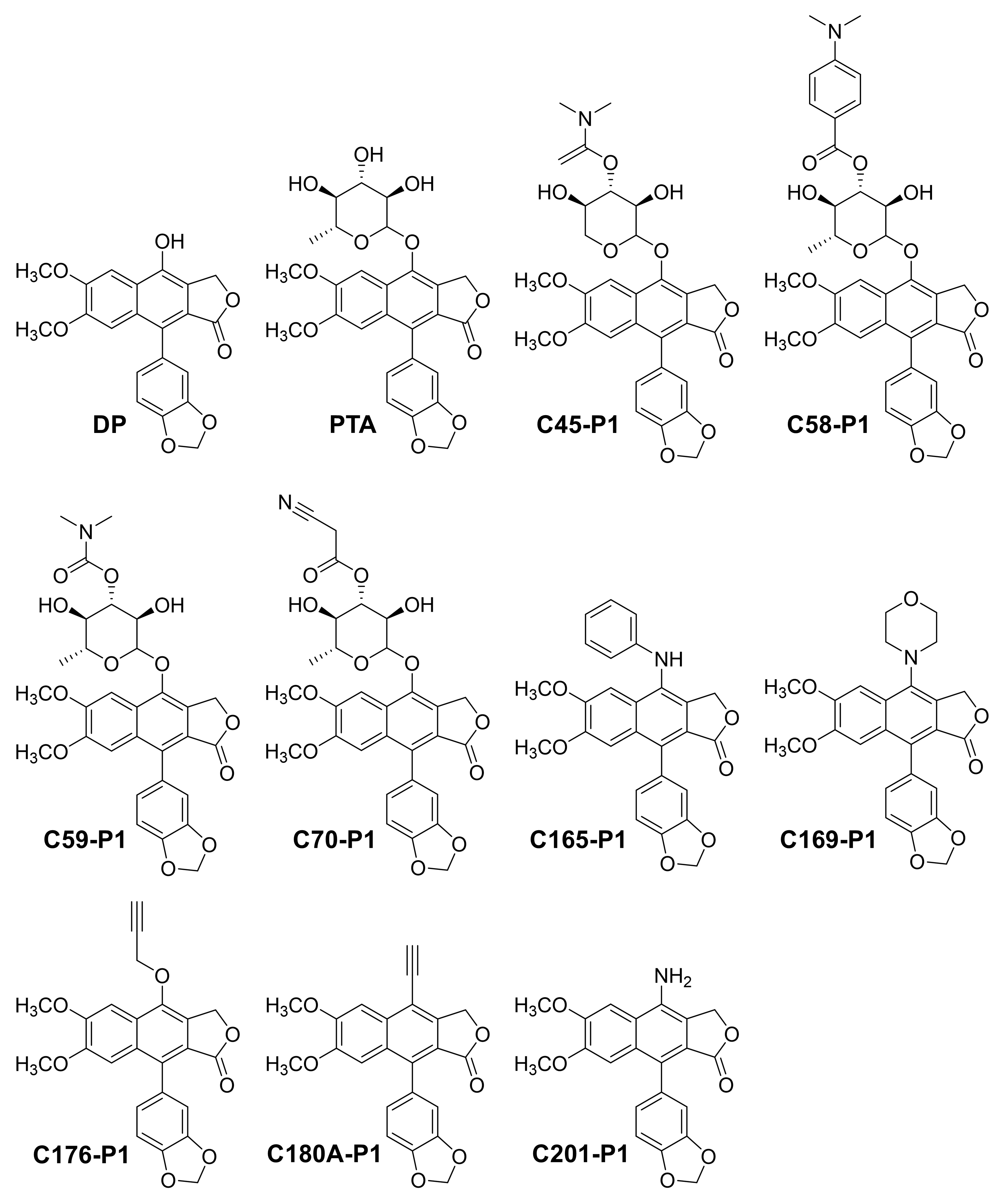
**

**Fig. S1.** The chemical structures of DP, PTA, DP derivatives (C176-P1 and C180A-P1), nitrogen-containing DP derivatives (C165-P1, C169-P1, and C201-P1), and nitrogen-containing PTA derivatives (C45-P1, C58-P1, C59-P1, and C70-P1).


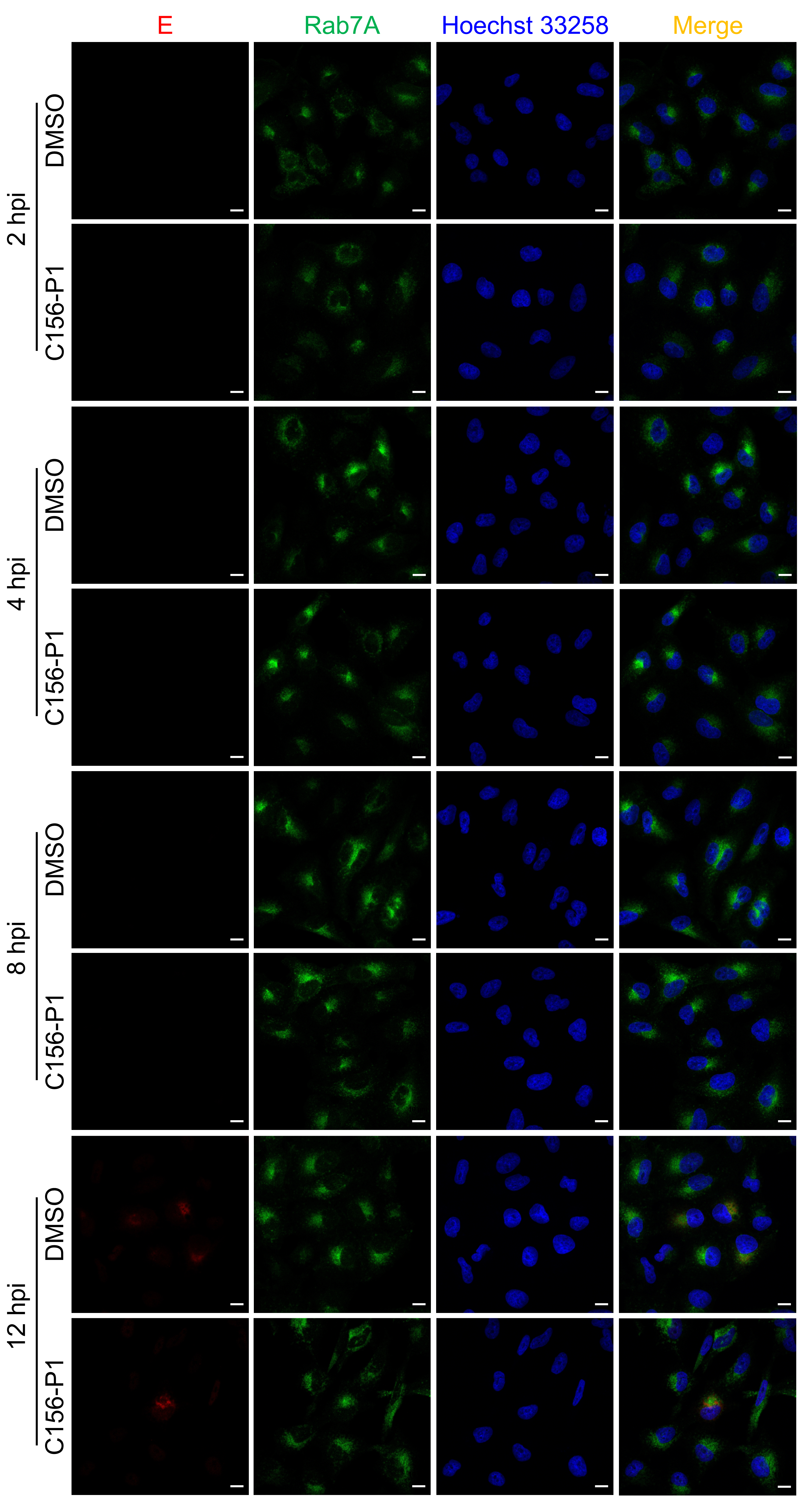


**Fig. S2.** DENV-2 E protein expression was examined at 2, 4, 8, and 12 hpi in A549 cells. A549 cells were transfected with plasmids expressing mNeonGreen-Rab7A for 24 h, followed by infected with DENV-2 (MOI = 20) and incubated with C156-P1 (50 nM) for 2 h, 4 h, 8 h, or 12 h. The cells were fixed and incubated with anti-DENV E primary antibodies, followed by Alexa Fluor 555-conjugated anti-Rabbit IgG secondary antibody for 1 h at room temperature. The representative images were acquired using the Zeiss LSM880. Scale bars: 10 μm.
